# A modern multimodal pain protocol eliminates the need for opioids for most patients following total knee arthroplasty: results from a retrospective comparative cohort study

**DOI:** 10.1186/s40634-023-00585-0

**Published:** 2023-02-20

**Authors:** Leland van Deventer, Amy Bronstone, Claudia Leonardi, Matthew Bennett, Peter Yager, Vinod Dasa

**Affiliations:** 1 School of Medicine, Louisiana State University Health Sciences Center, New Orleans, LA USA; 2 Department of Orthopaedic Surgery, Louisiana State University Health Sciences Center, 1542 Tulane Avenue, Box T6-7, New Orleans, LA 70112 USA; 3 School of Public Health, Louisiana State University Health Sciences Center, New Orleans, LA USA

**Keywords:** Opioids, Pain, Total knee arthroplasty, Multimodal analgesia, Retrospective cohort study

## Abstract

**Purpose:**

Modern multimodal analgesia has been shown to significantly reduce opioid use following total knee arthroplasty (TKA). This study was conducted to determine if changing TKA discharge opioid prescriptions from automatic to upon request resulted in more opioid free recoveries without compromising pain control.

**Methods:**

Between December 2019 and August 2021, an orthopedic surgeon performed 144 primary unilateral TKAs; patients received the same multimodal analgesia protocol except for postoperative opioid prescribing. The first consecutively-treated cohort automatically received an opioid prescription following discharge (automatic group) and the second cohort received opioid prescriptions only upon request (upon request group). Opioid prescription data were derived from a prescription monitoring program and patient-reported outcomes (PROs) were collected preoperatively and at 2 and 12 weeks postoperatively.

**Results:**

A higher percentage of the upon request group was opioid free 3 months after TKA compared with the automatic group (55.6% vs 4.3%, *p* < 0.0001) without compromising pain or function. Among opioid-naïve patients, 72% in the upon request group were opioid free after TKA compared with 5.4% in the automatic group. Opioid prescribing was not significantly reduced among opioid-experienced patients regardless of the pain protocol.

**Conclusion:**

Requiring patients to request opioid prescriptions following TKA resulted in a higher rate of opioid free TKA, especially among opioid-naïve patients, without increasing pain compared with offering all patients an initial opioid prescription.

**Level of evidence:**

Level III.

## Introduction

Despite efforts to reduce opioid prescribing, opioids continue to be commonly used for pain management after surgery in the United States (US), Canada, Australia, and some Western European countries [1–3]. During the year prior to total knee arthroplasty (TKA), more than one-third of patients in the US, Britain, and Sweden are prescribed opioids [1, 4]. Considerable evidence has amassed showing that excessive opioid prescribing following TKA is common in the US [5–9], and can lead to abuse, misuse, overdose, diversion, and dependance as well as an increased risk of TKA revision [10]. In addition, opioids have side effects, such as sedation, dizziness, nausea and vomiting, and constipation, that can hamper patient comfort and function following TKA [6, 11].

Multimodal analgesia, the use of different analgesics to address multiple pain receptors and pathways to improve pain relief while reducing individual class-related side effects, has been shown to significantly reduce opioid usage as a part of postoperative TKA analgesia [12–15]. Although multimodal analgesia has become the standard of care in TKA [13, 16, 17], there is no universally accepted regimen. In addition, a recent systematic literature review found a paucity of comparative studies that have evaluated interventions to achieve opioid-free recovery after hospital discharge following orthopedic surgery, including TKA [18].

The present comparative retrospective study was designed to determine whether a novel comprehensive multimodal analgesia regimen can allow an opioid-free recovery following TKA with no compromise in pain and function. It was hypothesized that patients who received opioids only upon request would use fewer opioids during the first three months after TKA and have equivalent patient-reported outcomes (PROs) compared to a group who received automatic opioids at discharge.

## Methods

### Design and treatment

The study was approved by the Louisiana State University Health Sciences Center- New Orleans (LSUHSC-NO) Institutional Review Board (Protocol #1561). This was a retrospective cohort study of consecutive patients who underwent primary unilateral TKA by a single fellowship-trained orthopedic surgeon between January 2019 and August 2021 using an enhanced recovery TKA protocol. Surgery for all patients consisted of a standard mechanical alignment protocol with computer-assisted TKA using Zimmer Biomet implants (NexGen® or Persona®, Zimmer Biomet Inc., Warsaw, IN, USA). All surgeries utilized an anterior midline incision and medial parapatellar arthrotomy.

All patients received the same multimodal pain protocol except for postoperative opioid prescribing. The first group of consecutively treated patients (January 2019 to December 2019) were automatically offered an opioid prescription upon hospital discharge (“automatic” group). The second group of consecutively-treated patients (May 2020 to August 2021) could receive an opioid prescription by requesting one from their attending physician (“upon request” group). Patients were excluded if they had undergone TKA for the contralateral joint or had TKA performed between January 2020 and March 2020, as traditional follow-up during this period was compromised due to the Covid-19 pandemic. No TKAs were performed between March 14, 2020, and May 1, 2020, due to Covid-19 restrictions. The upon request practice change started at the resumption of TKA cases following Covid-19 when inpatient hospital stays were prohibited, resulting in different patient selection criteria to reduce the likelihood of hospitalization.

The multimodal analgesia protocol was based on clinical evidence and extensive clinical experience. Approximately five days before TKA, all patients underwent percutaneous cryoneurolysis performed by the operating surgeon (VD) using the iovera device (iovera, Pacira CryoTech Inc., Fremont, CA) [19] after administering lidocaine for local analgesia. Cryoneurolysis involves applying cold (< 20 °C) to peripheral sensory nerves to induce Wallerian degeneration of nerve axons [20] and a long-acting nerve block [21], and has been shown to reduce opioid consumption following TKA [22–24]. The target of cryoneurolysis were the superficial geniculate nerves — the anterior femoral cutaneous nerve (AFCN) and the infrapatellar branch of the saphenous nerve (ISN) — which provide sensory innervation to the anterior knee and lie in a predictable and easily targetable location [25]. Immediately before undergoing surgery, patients received a single dose of 150 mg pregabalin, 200 mg celecoxib, and 1000 mg intravenous (IV) acetaminophen. An anesthesiologist performed neuraxial (spinal) anesthesia, consisting of 1.6 mg of 0.75% bupivacaine and occasional IV fentanyl depending on preferences of the anesthesia provider as well as an adductor canal block (ACB). Liposomal bupivacaine (Exparel®, Pacira Pharmaceuticals, Inc., San Diego, CA) was used for the ACB and was administered in the interspace between the popliteal artery and capsule of the posterior knee. Intraoperatively, a periarticular infiltration of 0.25% bupivacaine hydrochloride (Marcaine, Hospira, Inc., Lake Forest, IL) was administered.

Postoperatively, patients requiring overnight hospital admission received 650 mg of acetaminophen every 6 hours, 75 mg pregabalin twice daily, and 200 mg celecoxib twice daily. For patients requiring additional pain control prior to discharge, 1 mg of hydromorphone was typically administered. Upon discharge, all patients were advised to take 325 mg acetaminophen every four hours for two weeks and 75 mg diclofenac every 12 hours for 6 weeks.

For all patients in the study, the morphine milligram equivalents (MME) in the opioid prescription provided upon discharge was gradually decreased over time based on both the treating surgeon’s observation and patients’ feedback that fewer opioids were required to achieve sufficient pain control. For patients in the automatic group, the initial opioid prescription decreased from a high of 315 MME to a low of 105 MME. For the upon request group, the initial opioid prescription was decreased from a high of 140 MME to a low of 70 MME.

All patients could request additional opioids at any time point by contacting the surgeon’s office. All calls pertaining to pain and prescription of opioids were routed to the attending surgeon during business hours, and residents were not permitted to prescribe any opioids during call hours.

### Measures

Data collected from patients’ medical records included body mass index (BMI), overall deformity of the knee, laterality, Kellgren-Lawrence grade, prior TKA on contralateral knee, and demographic data (sex, age, race, insurance type, and surgery date). Opioid prescription data were obtained through the Louisiana Prescription Monitoring Program (PMP) database. Opioid prescriptions filled during the first three months before TKA were used to identify opioid-naïve (no opioid prescriptions) versus opioid-experienced (≥1 opioid prescription) patients. Data on opioid prescriptions filled during the first three months after TKA also were collected to derive key outcomes, including initial and refill opioid prescriptions, the MME of each prescription, and the provider who wrote the prescription.

PROs were evaluated pre-operatively and at 2 weeks and 3 months of follow-up. Pain was assessed using the Pain Intensity item from the Patient-Reported Outcomes Measurement Information System® (PROMIS-29®), which uses a 0–10 numerical pain rating scale, the PROMIS Pain Interference scale [26], and the Knee Osteoarthritis and Outcomes Score (KOOS) Pain subscale score [27]. Three additional KOOS scales were analyzed: Symptoms, Function in Daily life (ADL), and quality of life (QOL). The KOOS subscale raw scores were transformed into a 0–100 scale in which 0 indicates no problems and 100 extreme problems.

### Statistical analyses

Data were entered and managed using REDCap and analyzed using SAS version 9.4 (SAS Institute Inc., Cary, NC, USA). Baseline patients characteristics were compared between groups (automatic versus upon request) using the chi-square test for categorical variables with cell counts greater than five (sex, laterality, and opioid-naïve vs -experienced), the Fisher’s exact test for categorical variables with cell sizes less than 5 (race, health insurance, Kellgren-Lawrence grade and contralateral TKA), and the Student’s t-test for continuous normally distributed variables (age, BMI, overall deformity, and PROs).

Opioid prescriptions during first 3 months after TKA were compared between the two groups using the chi-square test when comparing proportions and Mann-Whitney U test when comparing medians. Median, minimum, and maximum values are reported for outcomes that were not normally distributed.

PROs at 2 weeks and 3 months were compared using repeated measures analysis of covariance to evaluate the effects of opioid prescription protocol, time, and the interaction of opioid prescription protocol by time while adjusting for age and pre-operative PROs. Insurance type was not included as a covariate as it was highly associated with age which was already included in the model. Since each PRO was collected multiple times for the same patient, the dependency between observations within patients was modeled using a compound symmetry covariance structure. Patients reported outcomes within each time point were compared between the two groups using the Student’s t-test. When implementing parametric models using a Normal distributions, residuals were independent and identically and normally distributed with homogenous variances indicating the assumptions were met. A two-sided alpha of less than 0.05 indicated statistical significance.

## Results

### Patient characteristics

A total of 144 patients were included (72 in each group). As shown in Table 1, the majority of patients were female (69.4%), white (59.0%), and opioid naïve (73.6%). Statistically significant differences between groups were observed for insurance type (*p* = 0.004) and age (p = 0.004), which were likely attributable to the provider’s practice transitioning to accept more Medicaid patients (four in the automatic and 16 in the upon request group), who tended to be younger, during the study. A larger percentage of patients were discharged the same day of surgery in the upon request versus automatic group (75.0% vs. 58.3%, *p* = 0.0339).

**Table 1:** Baseline Demographics and Clinical Characteristics

Characteristic	Automatic (***n*** = 72)	Upon Request (***n*** = 72)	***P*** value
Sex, % (n)			0.469
Male	33.3 (24)	27.8 (20)	
Female	66.7 (48)	72.2 (52)	
Race, % (n)			0.257
Black or African American	37.5 (27)	33.3 (24)	
White or Caucasian	54.2 (39)	63.9 (46)	
Other	8.3 (6)	2.8 (2)	
Health Insurance, % (n)			0.004
Private	36.1 (27)	45.8 (33)	
Medicare	18.0 (13)	9.7 (7)	
Medicaid	5.6 (4)	22.2 (16)	
Medicare Advantage	34.7 (25)	22.2 (16)	
Other	5.6 (3)	0 (0)	
Kellgren-Lawrence grade,% (n)^a^			0.127
2	0 (0)	1.4 (1)	
3	5.6 (4)	13.9 (10)	
4	94.7 (67)	84.7 (61)	
Laterality, % (n)			0.739
Right	45.8 (33)	48.6 (35)	
Left	54.2 (39)	51.4 (37)	
Opioid-naive, % (n)	77.8 (56)	69.4 (50)	0.257
Contralateral TKA			0.228
None	79.2 (57)	90.3 (65)	
Within 6 months	6.9 (5)	2.8 (2)	
After 6 months	13.9 (10)	6.9 (5)	
Age (years), mean (SD)	69.5 (7.9)	65.2 (9.4)	0.004
BMI (kg/m^2^), mean (SD)	33.9 (6.4)	32.5 (6.0)	0.165
Overall deformity (°), mean (SD)	9.0 (4.9)	8.9 (5.5)	0.921
KOOS, mean (SD)			
Pain	36.3 (20.4)	33.8 (20.1)	0.475
Symptoms	40.6 (21.5)	37.4 (20.8)	0.364
ADL	39.3 (22.4)	36.8 (21.4)	0.497
QOL	19.5 (16.6)	19.5 (20.0)	0.980
PROMIS-29, mean (SD)			
Pain Interference	65.0 (9.1)	66.0 (6.9)	0.465
Pain	7.1 (2.2)	7.5 (2.3)	0.285

^a^Data were missing for one patient who did not have a preoperative x-ray

*ADL* Function in daily living, *BMI* Body mass index, *KOOS* Knee injury and Osteoarthritis Outcome Score, *PROMIS* Patient-Reported Outcomes Measurement Information System, *QOL* Quality of life, *SD* Standard deviation

### Opioid-free TKA

As shown in Table 2, during the first three months after TKA, 55.6% of patients in the upon request group did not receive any opioids during the three months after TKA compared with 4.2% of the automatic group (*p* < 0.0001). Among opioid-naïve patients, 72.0% in the upon request group did not receive any opioids during the first three months after TKA compared with 5.4% of the automatic group (*p* < 0.0001). In contrast, among opioid-experienced patients, 18.2% of patients in the upon request group did not receive any opioids compared with 0% of the automatic group (*p* = 0.124).

**Table 2:** Opioid Prescriptions During First 3 Months After TKA

Outcome^a^	Automatic (***n*** = 72)	Upon Request (***n*** = 72)	***P*** value
All Patients			
≥ 1 filled opioid prescription, % (n)	95.8 (69)	44.4 (32)	< 0.0001
No. of prescriptions	1 (0–8)	0 (0–8)	< 0.0001
≥ 1 refill, % (n)	43.1 (31)	25.0 (18)	0.022
Patients with ≥1 Opioid Prescription			
No. of prescriptions	1 (1–8)	2 (1–8)	0.111
Patients with ≥1 Opioid Refill			
No. of refills	2 (1–7)	3 (1–6)	0.093
Opioid-naïve Patients, n	56	50	
≥ 1 filled opioid prescription, % (n)	94.6 (53)	28.0 (14)	< 0.0001
No. of prescriptions	1 (0–8)	0 (0–7)	< 0.0001
≥ 1 refill, % (n)	33.9 (19)	10.0 (5)	0.003
Opioid-naïve patients with ≥1 Opioid Prescription			
No. of prescriptions	1 (1–8)	1 (1–7)	0.704
Opioid-naïve patients with ≥1 Opioid Refill			
No. of refills	3 (1–6)	3 (1–7)	0.823
Opioid-experienced Patients, n	16	22	
≥ 1 filled opioid prescription, % (n)	100 (16)	81.8 (18)	0.124
No. of prescriptions	3 (1–7)	2 (0–8)	0.352
≥ 1 refill, % (n)	75.0 (12)	59.1 (13)	0.490
Opioid- experienced patients with ≥1 Opioid Prescription			
No. of prescriptions	3 (1–7)	2.5 (1–8)	1.000

^a^All values are median (range) unless otherwise specified

*MME* Morphine milligram equivalent, *TKA* Total knee arthroplasty

### Opioid prescriptions by orthopedic team or another provider

**Fig. 1:**
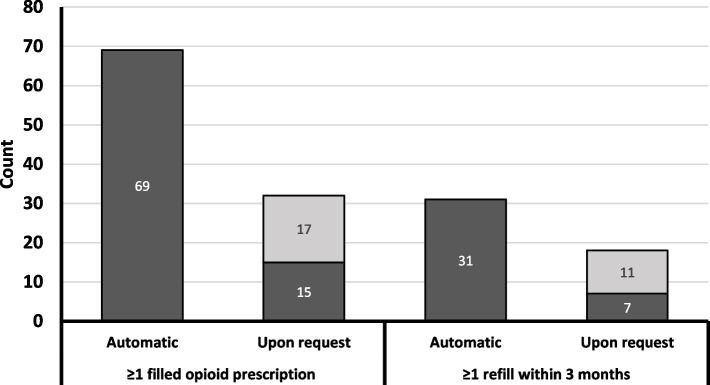
Number of Patients with at Least One Initial and Refill Opioid Prescription During the First 3 Months After TKA. Dark gray indicates patients whose opioids were prescribed by a member of the orthopedic team and light gray indicates patients whose opioids were prescribed another provider

Figure 1 shows the number of patients who received an initial or a refill opioid prescription by provider type (orthopedic team member versus another provider). In the automatic group, all initial and refill opioid prescriptions were written by a member of the orthopedic team. In contrast, in the upon request group, 53.1% (17 of 32) of initial opioid prescriptions and 61.1% (11 of 18) of refills were written by another provider.

Among all patients and opioid-experienced patients, those who obtained prescriptions from another provider had a higher median MME for the initial opioid prescription (all patients: 225 vs. 75, *p* = 0.001; opioid-experienced: 300 vs. 75, *p* = 0.004) and higher MME for all opioid prescriptions (all patients: 560 vs. 150, *p* = 0.013; opioid-experienced: 975 vs. 225, *p* = 0.023) than patients who received opioid prescriptions from the orthopedic team. Among opioid-naïve patients, there were no statistically significant differences in the median MME for the initial opioid prescription (140 vs. 90, *p* = 0.098) and all prescriptions (145 vs. 140, *p* = 0.552) between those receiving prescriptions from other providers versus the orthopedic team.

### Patient-reported outcomes

There were no statistically significant differences between groups on any PRO measure at 2 weeks or 3 months after TKA (Table 3). As shown in Table 4, both groups significantly improved on all PROs from the preoperative visit to the 3-month follow-up visit. There were no statistically significant differences between groups in any of the PRO measures at 3-month follow-up. There was a statistically significant treatment by time interaction for the KOOS Symptoms scale such that the automatic group had significantly more improvement over time than the upon request group.

**Table 3:** Patient-reported Outcomes at 2 Weeks and 3 Months After TKA by Treatment Group

Outcome^a^	n	Automatic	Upon Request	***P*** value
KOOS Pain				
2 weeks	92	48.2 (2.6)	46.7 (2.8)	0.712
3 Months	84	66.6 (3.6)	61.1 (3.3)	0.191
KOOS Symptoms				
2 weeks	93	48.6 (2.4)	53.9 (2.6)	0.139
3 Months	84	65.5 (2.4)	59.3 (3.1)	0.113
KOOS ADL				
2 weeks	91	53.5 (2.7)	50.6 (2.9)	0.482
3 Months	85	69.9 (2.6)	66.3 (3.3)	0.393
KOOS QOL				
2 weeks	90	30.6 (2.9)	34.8 (3.1)	0.335
3 Months	83	49.9 (2.8)	42.7 (3.7)	0.123
PROMIS-29				
Pain Interference				
2 weeks	82	63.2 (1.3)	65.5 (1.2)	0.199
3 Months	79	55.4 (1.2)	56.4 (1.4)	0.600
Pain Intensity				
2 weeks	88	5.4 (0.4)	5.9 (0.4)	0.352
3 Months	83	3.8 (0.4)	3.6 (0.4)	0.676

^a^All values are least squares means (standard error of the mean) adjusted for age and pre-operative score

*ADL* Activities of daily living, *KOOS* Knee injury and Osteoarthritis Outcome Score, *PROMIS* Patient-Reported Outcomes Measurement Information System, *QOL* Quality of life, *TKA* Total knee arthroplasty

**Table 4:** Patient-reported Outcomes 3 Months After TKA

Outcome^a^	Automatic (***n*** = 72)	Upon Request (***n*** = 72)	Fixed effects		
			Group	Time	Group*Time
KOOS			---------- *p*-value ----------		
Pain	57.4 (1.8)	53.9 (2.2)	0.226	< 0.0001	0.480
Symptoms	57.0 (1.7)	56.6 (2.0)	0.864	< 0.0001	0.031
ADL	61.7 (1.9)	58.5 (2.2)	0.270	< 0.0001	0.890
QOL	40.2 (2.0)	38.7 (2.4)	0.635	< 0.0001	0.075
PROMIS-29					
Pain Interference	59.3 (0.9)	60.9 (0.9)	0.204	< 0.0001	0.603
Pain	4.6 (0.3)	4.8 (0.3)	0.731	< 0.0001	0.342

^a^All values are least squares means (standard error of the mean) adjusted for age and pre-operative score

*ADL* Activities of daily living, *KOOS* Knee injury and Osteoarthritis Outcome Score, *PROMIS* Patient-Reported Outcomes Measurement Information System, *QOL* Quality of life

## Discussion

The novel multimodal analgesia regimen used in this study resulted in approximately 7 of 10 opioid-naïve patients achieving an opioid-free TKA while experiencing adequate pain control, suggesting the goal of opioid-free TKA is very realistic for most opioid-naïve patients. Consistent with our study, another recently published study reported very low opioid use among 386 prospective, consecutive TKA patients (356 of whom were opioid naïve and 30 were opioid experienced) who received a 4-month protocol that included multimodal analgesia, education, goal setting and realistic expectations, shared decision making, and a simplified physical therapy protocol designed to decrease post-operative pain and swelling [28]. Among the opioid-naïve patients in this study, 73/356 (20%) used opioids postoperatively, which is very similar to the rate of 18% in the present study. Further, opioid use was very low among all 386 patients with 86.3% using 10 or fewer opioid pills through 3 months postoperatively [29]. Together, our findings and these data suggest that achieving opioid-free or opioid-minimal TKA may be possible in both opioid-naïve and opioid-experienced patients by expanding the window of multimodal pain management from the first 72 hours after surgery to 3 months after TKA and prescribing a low amount of opioids [28].

The major challenges indicated by our data are the difficulties of reducing opioid use among opioid-experienced patients and controlling opioid prescribing among providers outside the orthopedic team. Acute pain management in opioid-tolerant and-dependent patients is a complex issue because using opioids sparingly may result in both poor pain management and withdrawal. Tolerance to opioids, in which exposure makes patients less susceptible to opioids’ therapeutic effects [30], can develop within 1 week [31], with tolerant patients requiring higher doses of opioids after surgery to effectively manage their pain. Chronic opioid use prior to TKA may lead to physical dependence, a state of adaptation that produces symptoms of withdrawal when the drug is abruptly stopped or dose rapidly reduced [30]. A recent study evaluated the effectiveness of a transitional pain service intervention aimed at completely tapering chronic opioid users off opioids by 60 days after elective surgery [32]. Impressively, 70% of chronic opioid users who underwent orthopedic surgery completely tapered off opioids within 60 days after discharge from the hospital and experienced significant improvements in pain intensity and interference compared to patients who did not completely cease opioids. Additional research is needed to determine whether similar programs can help opioid-experienced TKA patients taper their use after surgery.

A noteworthy finding was that providers outside of the orthopedic team wrote opioid prescriptions for much higher amounts than providers within the orthopedic team. This result is consistent with findings from Namba et al. that orthopedic surgeons wrote only 47% of opioid prescriptions for TKA patients during the first 3 months postoperatively, which decreased to 14% from nine to 12 months postoperatively [33]. General medicine and internal medicine physicians were the two most likely specialties to write opioid prescriptions both during the year before and after TKA [33]. Similarly, in a study of patients with knee osteoarthritis, non-orthopedic physicians, who were responsible for 92% of all opioid prescriptions, prescribed a greater number of opioid prescriptions per patient, higher dosages, and more refills than orthopedists [34]. To reduce opioid prescribing among non-orthopedists both prior to and after TKA, non-orthopedic providers should consider earlier referral to an orthopedic surgeon for management of patients with end-stage knee osteoarthritis for whom non-opioid conservative management is not effective and should require referral of postoperative TKA patients with increased orthopedic-related analgesic requirements to their surgeon’s practice for evaluation and further care. For example, to improve communication among providers at one institution, TKA patients and their primary care providers receive a letter when the patient is about to be admitted for surgery informing patients that they are to receive opioid prescriptions only from their surgeon during the first 90 days after surgery with the expectation that they will be weaned off opioids by 6–12 weeks postoperatively [33].

The main limitations of this study are its retrospective nature, lack of randomization by opioid protocol (automatic vs. upon request), sample size, potential influence of selection bias, potential confounding effects of reducing the initial opioid prescription MME, and involvement of a single orthopedic surgeon practicing at a single institution in the US. Due to Covid-19, patients in the upon request group were selected for being less likely to require hospitalization and this group was more likely to be discharged on the same day as surgery compared with the automatic group. Although this raises the possibility that selection bias may have affected results, we note that the two groups were similar with respect to variables more likely to influence post-discharge opioid use, such as preoperative opioid use and baseline self-reported pain. However, we acknowledge the possibility that the automatic group differed from the upon request group in ways we were unable to measure which resulted in the latter group having lower opioid requirements. Treatment by a single orthopedic surgeon at a single site in the US limits the generalizability of findings, which should be replicated in other settings, preferably using a randomized, parallel-group design. In addition, findings are most applicable to countries that commonly utilize opioids as part of post-operative pain control. The MME for the initial prescription was gradually reduced over time in both groups, such that, among all patients who received an initial opioid prescription, the MME was significantly lower in the upon request group than automatic group; because lowering the opioid dose confounds this finding, differences in MMEs between groups have not been presented or interpreted. Despite being provided fewer opioids initially, we note that the upon request group was less likely to request a refill opioid prescription and reported similar pain and function as the automatic group, indicating that lowering the initial opioid prescription was well tolerated and that opioid-free recovery is possible for the majority of opioid-naïve TKA patients. An additional limitation is that we measured opioid prescriptions filled rather than opioids consumed. It is possible that patients in the automatic group received an opioid prescription but did not use any opioids or used very few opioids. Lastly, we did not evaluate the specific medical condition for which patients had been prescribed opioids prior to or after TKA.

## Conclusion

More than half of all TKA patients and 72% of opioid-naïve patients who were treated with a multimodal pain protocol designed to minimize opioid use for up to 3 months, and received opioids only upon request after surgery, recovered from TKA without the use of any opioids and any worsening of self-reported pain or knee-related problems compared with patients who received an opioid prescription automatically upon discharge. The remaining main challenges are to ensure that opioids are tapered in patients who use them postoperatively and to better control opioid prescribing among non-orthopedic providers both before and after TKA.
